# 2814. The Impact of Novel Antibiotics on Curbing Antimicrobial Resistance and Infection Spread a Dynamic Modeling Study

**DOI:** 10.1093/ofid/ofad500.2425

**Published:** 2023-11-27

**Authors:** Apoorva Ambavane, Michal Litkiewicz, Aditya Sardesai, Diana Teloian, Engels N Obi, Joe Yang

**Affiliations:** Evidera, London, England, United Kingdom; Evidera, London, England, United Kingdom; Evidera, London, England, United Kingdom; Evidera Inc., Budapest, Budapest, Hungary; Merck & Co., Inc, BASKING RIDGE, New Jersey; Merck Co., Upper Gwynedd, Pennsylvania

## Abstract

**Background:**

Antimicrobial resistance (AMR) is a growing global concern. Novel antibiotics (ABXs) target resistant pathogens causing gram-negative (GN) bacterial infections. This study assesses the broader impact of novel ABX use on the US healthcare system, including preventing the onward spread of infections and reducing the level of high-threat AMR.

**Methods:**

A stochastic dynamic transmission model was developed to simulate the interaction between infection transmissibility, bacterial resistance development, and multiple lines of ABX. Included infection types were intra-abdominal infections, urinary tract infections and hospital-acquired /ventilator-associated pneumonia. Included AMR types were carbapenem-resistant and ESBL-producing enterobacterales (CRE and ESBLE), and multi-drug resistant pseudomonas aeruginosa (MDRP). The model has compartments for non-infected, colonized, or infected patients, with a sensitive or resistant strain in a US hospital. Infected patients receive antibiotics resulting in infection clearance or use of an alternate ABX class. Sensitive pathogens gain resistance due to drug pressure or conversely could lose resistance. Ordinary differential equations were used to estimate compartment membership. The primary data source was the Premier healthcare database (172 hospitals, 2016-2020). The model examined the potential reduction in AMR levels and avoided hospital infections, by comparing two scenarios: targeted use of novel ABX for specific AMR types and the status quo with limited novel ABX use.

**Results:**

When even novel ABXs are reserved as the 2^nd^ or 3^rd^ line of treatment, compared to the status quo, it could result in a ∼1.5% reduction in hospital infections and a change in CRE, ESBLE, and MDRP by −2.5%, −3.6%, and −2.1%, respectively, on an annual basis. The cumulative impact of novel ABXs over 5-10 years was much larger but subject to higher prediction uncertainty. The primary driver of results are infection clearance rates, risk of resistance gain on various ABX, and uptake of novel ABX.

**Conclusion:**

Targeted use of novel ABXs for resistant pathogens could lead to improved, earlier infection clearance with most types of infections and pathogens, thereby reducing infection transmission and rise of high-threat AMR in the long-term.

**Disclosures:**

**Apoorva Ambavane, PhD**, Merck & Co., Inc: Grant/Research Support **Michal Litkiewicz, MSc**, Merck & Co., Inc: Grant/Research Support **Aditya Sardesai, MSc**, CSL Seqirus: Advisor/Consultant|Merck & Co. Inc: Grant/Research Support|Merck & Co. Inc: Grant/Research Support **Diana Teloian, MA**, Merck & Co., Inc: Grant/Research Support **Engels N. Obi, PhD**, Merck & Co Inc: Employee **Joe Yang, PhD**, Merck & Co., Inc: Employee|Merck & Co., Inc: Stocks/Bonds

